# Novel delivery systems for phages and lysins in the topical management of wound infections: a narrative review

**DOI:** 10.3389/fmicb.2025.1526096

**Published:** 2025-01-27

**Authors:** Pan Yang, Jing Li, Xiumei Ma, Nan Hu, Zhangyong Song, Bin Chen, Shizhu Li

**Affiliations:** ^1^Postdoctoral Research Station, Guangzhou Bay Area Institute of Biomedicine, Guangzhou, China; ^2^School of Basic Medical Sciences, Southwest Medical University, Luzhou, China; ^3^Sichuan Provincial Key Laboratory for Human Disease Gene Study and Department of Laboratory Medicine, Sichuan Provincial People’s Hospital, University of Electronic Science and Technology of China, Chengdu, China; ^4^Department of Dermatology, Southwest Hospital, Third Military Medical University (Army Medical University), Chongqing, China; ^5^State Key Laboratory of Ultrasound in Medicine and Engineering, College of Biomedical Engineering, Chongqing Medical University, Chongqing, China

**Keywords:** wound, phage, lysin, multidrug-resistant, therapy

## Abstract

Currently, multidrug-resistant (MDR) bacterial wound infections (WIs) are an extremely challenging clinical problem for physicians. Recently, compared to traditional single liquid delivery drugs, the study of five novel drug delivery systems (i.e., hydrogel, liposomes, electrospun fibers, nanoparticles and nanoemulsion) for phages and their encoded lysins in WI management has become a hot topic. To assess the current landscape of these emerging technologies, we conducted a comprehensive literature search across PubMed, Scopus and Web of Science up to July 2024, using terms such as “phage,” “lysin,” “wound,” “hydrogel,” “liposomes,” “fibers,” “nanoparticles,” and “nanoemulsion.” The criteria included original studies of five novel delivery systems for phages and lysins in WI management. The findings highlighted the positive effects of the five novel delivery systems for phages and lysins in WI management, significantly reducing wound bacterial populations, and accelerating healing at the injury site. However, the available literature on novel delivery systems for phages and lysins remains limited, particularly for lysins. In conclusion, the application of novel drug delivery systems for phages and lysins showed great potential in combating MDR bacterial WIs.

## 1 Introduction

Currently, the treatment of multidrug-resistant (MDR) bacterial wound infections (WIs) faces significant challenges. The American Academy of Dermatology reported that the total number of patients seeking treatment for burns, ulcers, wounds and skin infections in the U.S. exceeded 50 million in 2013, and the total cost of treatment was approximately $19.9 billion (American Academy of Dermatology Association, 2018). With the considerable overuse of antibiotics to treat WIs, the emergence of MDR bacterial WIs has become a headache for clinicians and the public health system. Due to the tightening of financial strategies of global pharmaceutical companies and slow progress in antibiotic development, the search for alternatives to antibiotics to treat MDR bacteria has become particularly urgent (Leptihn, 2019; Manohar et al., 2020).

In recent years, phage therapy and phage-encoded lysins as new antimicrobial agents provide the revitalied weapons to address the antibiotic resistance dilemma (Dedrick et al., 2019; Sweere et al., 2019; Rahman et al., 2021; Feng et al., 2024). Phages use bacteria as parasitic hosts, proliferate inside the host bacteria and release them outside the host bacteria, resulting in bacterial lysis and death. Generally speaking, the phage life cycle is as follows (Briot et al., 2022; Liang et al., 2023): (1) Adsorption: the phage achieves tight adsorption to the host bacteria through the specific binding of its tail fibers to the surface of the host bacteria. (2) Invasion: after tightly adhering to the host bacteria, the phage injects its genes into the host bacteria. (3) Proliferation: the phage uses structural components of the host bacteria to synthesize progeny phage proteins and nucleic acids. (4) Assembly: the synthesized phage components are assembled together according to a certain procedure. (5) Release: upon completion of assembly, the phage cleaves the cell wall through its secreted lytic enzyme, and then releases it into the extracellular space of the host bacteria. Through the repeated cycles of the above steps, the phage kills a large number of host bacteria, thus exerting its powerful bactericidal effect. Therefore, microbiologists and clinicians often isolate and screen phages from natural environments to produce phage preparations for antimicrobial treatments known as phage therapy. In fact, phages rely mainly on their derived enzymes to exert their antibacterial properties. As one of the most important phage-derived enzymes, endolysin kills bacteria by degrading the peptidoglycan structure of the bacterial cell wall during phage-mediated internal lysis –“endo” meaning “inside”-which occurs at the end of the phage infection cycle (Rahman et al., 2021; Chang et al., 2022). Based on the above description, attention in this review is therefore focussed on the research of endolysins (lysins). Although phages are obtained from natural rapid isolation, phage-derived lsyins need to be produced by recombination in a bacterial or yeast expression system. With the rapid development of genetic engineering, large quantities of purified phage-derived lysins can be readily obtained, thus opening up the possibility of large-scale clinical therapy.

The research of phage therapy as an alternative to conventional antibiotics in the treatment of MDR-refractory WIs such as dermatoses, burns and chronic wound/ulcer infections is again of great interest to traumatologists worldwide (Ding et al., 2022; Wang et al., 2024). Early phage therapy for the treatment of superficial bacterial infections, particularly dermatological diseases, burns and chronic wound/ulcer infections, attracted considerable attention. Despite the excellent antibacterial efficacy of phage therapy, phage therapy only has antibacterial properties against a single pathogenic bacteria, which is one of the reasons why phage therapy was difficult to be applied on a wide clinical scale at that time. Soon after, with the discovery of broad-spectrum antibiotic penicillin, phage therapy came to an abrupt end in most Western countries. Surprisingly, phage therapy is still in use at institutions such as Hirszfeld (Wrocław, Poland) and Eliava (Tbilisi, Georgia). Nowadays, phages or their derived lysins modified by genetic engineering and *in vivo* recombinant engineering can remove unwanted properties, thus potentially altering specificity or improving therapeutic potential. Peng et al. (2022) replaced the receptor-binding structural domain of phage M13 (the N-terminal structural domain of g3p) with the corresponding structural domain of phage Pf1, which enabled the chimeric M13-g3p (Pf1) to (Pf1) to attach to *Pseudomonas aeruginosa* (*P. aeruginosa*) via type IV pili. Moreover, there has been an increasing interest in improved lysins with new properties. Modified lysins are formed by binding of natural lysin fragments to other proteins or by rearrangement of the catalytic N-terminal structural domain and cell wall binding structural domain. The fusion of an outer membrane permeable peptide or cationic peptide with phage lysins can sensitize Gram-negetive (G^–^) bacteria to its lysogenic effect or enhance the bactericidal effect against *Streptococcus* spp. (Briers et al., 2014a; Briers et al., 2014b; Rodriguez-Rubio et al., 2016). Yang et al. (2015) constructed a novel modified lysin, ClyR, by fusing the CHAP structural domain of the PlyC lysin to the CBD of the PlySs2 lysin. It has potent bactericidal activity and a broad streptococcal host range, including a wide variety of *streptococci* as well as representative *enterococci* and *staphylococci* (including *MRSA* and *VRSA*). Based on the above, phage and lysin therapies for WIs caused by MDR bacteria are once again showing a very tempting prospect, along with the continuous modification of phages and lysins and the emergence of MDR bacteria (De Maesschalck et al., 2020; Prokopczuk et al., 2023; Karn et al., 2024).

**FIGURE 1:**
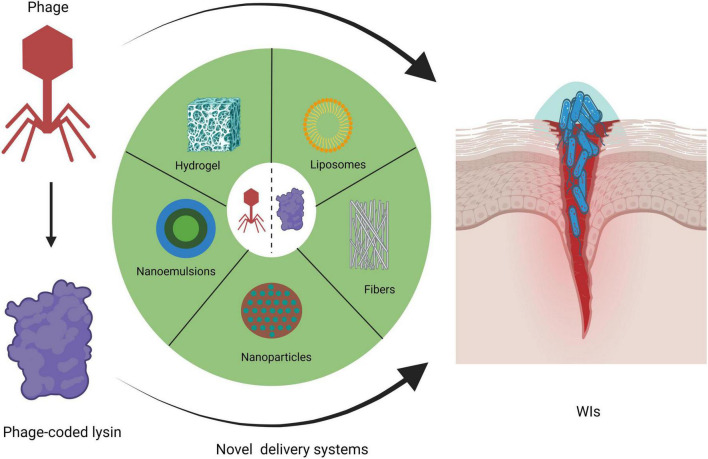
Novel delivery systems for phages and lysins in topical WI management (created by BioRender.com, 2024).

Hitherto, the U.S. Food and Drug Administration has granted consecutive approvals for the phage cocktail Sb-1, and the phage-derived lysins N-Rephasin^®^ SAL200, and CF-301 for use in clinical emergencies and special situations. Staphefekt SA.100 is designated as a Class 1 medical device in Europe for the treatment of superficial skin lesions infected with *Staphylococcus aureus (S. aureus*) (de Wit et al., 2019). The best route of administration for the phage and its encoded lysin for the treatment of WIs is likely to be topical to ensure that the drug is delivered directly to the infected wound site, thus avoiding its destruction by the host immune and digestive systems. Topical administration is a safer, more cost-effective and convenient route of administration compared to intravenous and oral administrations. Table 1 summarizes the current clinical trial landscape of phage and lysin formulations for the treatment of WIs. It can be seen that liquid or creams is the main delivery mode of phage and lysin formulations for the treatment of WIs. Liquid phage preparations are usually formulated in sterile buffer solutions such as phosphate buffered saline (PBS) or Tris-buffered saline-magnesium buffer (SMB) (Chang et al., 2020). As a result, liquid phage formulations are simple to prepare and relatively minimal formulation development is required for phage stability (Chang et al., 2020). Liquid preparations are usually impregnated in dressings, gauze and bandages for covering wounds (Pinto et al., 2020; Steele et al., 2020). However, phages may become stuck in the gauze, hindering phage release and subsequent bacteriolysis. In an impressive PhagoBurn study, jointly organized by France, Belgium and Switzerland, the efficacy of phage cocktail (PP1131) in the treatment of burn wounds failed to meet expectations, mainly due to the rapid decrease in the total titre of the phage cocktail to 10^4^–10^5^ PFU/mL over a period of 6 months (Jault et al., 2019). The instability of PP1131 may be due to electrostatic interactions, adsorption on the surface of the storage container, oxidation, or water chemistry interference (Jault et al., 2019; Mutti and Corsini, 2019; Chang et al., 2020). In addition, thicker phage cream preparations may have added ingredients that can limit phage movement, but the presence of preservatives in creams, especially those with an acidic pH, can negatively affect phage effectiveness (Merabishvili et al., 2017; Pinto et al., 2020). Cream delivery of lysins also does not perfectly improve the survival of lysins at pH values close to 5 and is thermally triggered at temperatures similar to those of infected wounds (Pinto et al., 2021). This makes it difficult to obtain satisfactory stability, long shelf-life and definitive therapeutic effect of lysin cream formulations (Pinto et al., 2021). To date, a number of novel delivery systems (e.g., hydrogel, liposomes, electrospun fibers, nanoparticles and nanoemulsions) for the delivery of phages and lysins have received more and more keen attention from scholars, as illustrated in Figure 1 (Chang et al., 2020; Pinto et al., 2020; Pinto et al., 2021; Briot et al., 2022). As far as the currently available data are concerned, there has not yet been a comprehensive and detailed review on the use of these novel delivery systems for phages and lysins in the topical management of WIs. Here, we will focus on an overview of the literature on these five novel drug delivery systems for the treatment of WIs, with a view to providing important implications for researchers working in this field.

**TABLE 1:** Summary data of clinical trials of phage and lysin therapy for WIs.

Application	Delivery approach	Registration date	Phase	Clinical trial registration number	Study status	Results
**Clinical trials of phages**						
Venous leg ulcers	Liquid	Apr 22, 2008	Phase 1	NCT00663091	Completed	This study found no safety concerns with the bacteriophage treatment.
Burn wound	Liquid	Dec 11, 2012	Phase 1/2	NCT02116010	Forced to end early, and related research published	The efficacy of phage therapy is not as expected. The only positive outcome of the trial was that it resulted in fewer adverse reactions compared to the control group.
DFU	Liquid	Jan 27, 2016	Phase 1/2	NCT02664740	Completed	Phage therapy showed safe and effective in treating 42 patients with chronic venous leg ulcers using *P. aeruginosa*, *S. aureus*, and *E. coli* phages.
Burn WIs	Liquid spray	Mar 20, 2020	Phase 1	NCT04323475	Recruiting	Not provided.
DFU	Liquid	Mar 2, 2021	Phase 1/2	NCT04803708	Completed	Not provided.
Pressure ulcers	Liquid spray	Mar 19, 2021	Phase 1/2	NCT04815798	Recruiting	Not provided.
Surgical WIs	Liquid	Mar13, 2024	Phase 1/2	NCT06319235	Recruiting	Not provided.
Open WIs	Liquid	May 9, 2024	Phase 1/2	ChiCTR2400084025	Recruiting	Not provided.
**Application**	**Formulation type**	**Registration date**	**Phase**	**Clinical trial registration number**	**Study status**	**Results**
**Clinical trials of lysins**						
AD	Creams	Jul 21, 2016	Phase 1/ 2	NCT02840955	Completed.	Lysin treatment had a significant advantage in the number of recurrencles of AD during the intervention period. Long-term targeted lysin therapy against *S. aureus* was well tolerated in AD patients but did not reduce topical corticosteroids use. Phase III clinical trials are needed to provide further strong evidence of topical lysin versus antibiotics for the treatment of *S. aureus* superficial skin infections.

## 2 Methods

### 2.1 Search strategy

Three electronic databases were searched, without limits, for articles published up to 25th July 2024: the PubMed, Scopus and Web of Science databases. The search was performed using the following terms: “phage” AND “hydrogel” OR “liposomes” OR “fibers” OR “nanoparticles” OR “nanoemulsion” AND “wound” or “lysin” AND “hydrogel” OR “liposomes” OR “fibers” OR “nanoparticles” OR “nanoemulsion” AND “wound.”

### 2.2 Study inclusion and exclusion criteria

The selection of studies for inclusion was limited to original research, particularly *in vitro* and *in vivo* animal experimental studies on infected wounds. The exclusion criteria were strictly defined to ensure relevance and quality; we omitted reviews, short communication, encyclopedias, editorials, conference information, conference abstracts and book chapters. The first step in the research methodology was to identify and exclude duplicate study using Endnote (version X8) from the database to avoid redundancy. Moreover, additional literature was included to enrich the manuscript and deepen the thematic structure of the article during the manuscript writing process. Title and abstract screening was performed independently by two authors (PY, Xiumei Ma), with discrepancies resolved by a third author (NH). Full-text screening was performed independently by two authors (PY, Xiumei Ma), with discrepancies resolved by a third author (NH).

### 2.3 Data extraction

The following information was extracted from each study: authors; date of publication; location of the study; type of wound; synthetic material for the novel delivery system; causative organisms; phage and lysin names and their dosages; type of study design; therapeutic efficacy (including change in bacterial counts in wounds, survival in mice, wound contraction, wound tissue healing, and change in inflammation in wound tissue). Comments or data on the safety, stability, and adverse effects of the formulations were also collected. Data were extracted independently by two authors (PY, Xiumei Ma), and inconsistencies were resolved by a third author (NH). This review was conducted in accordance with the PRISMA (Preferred Reporting Items for Systematic Reviews and Meta-Analyses) guidelines (Stroup et al., 2000).

## 3 Results

**FIGURE 2:**
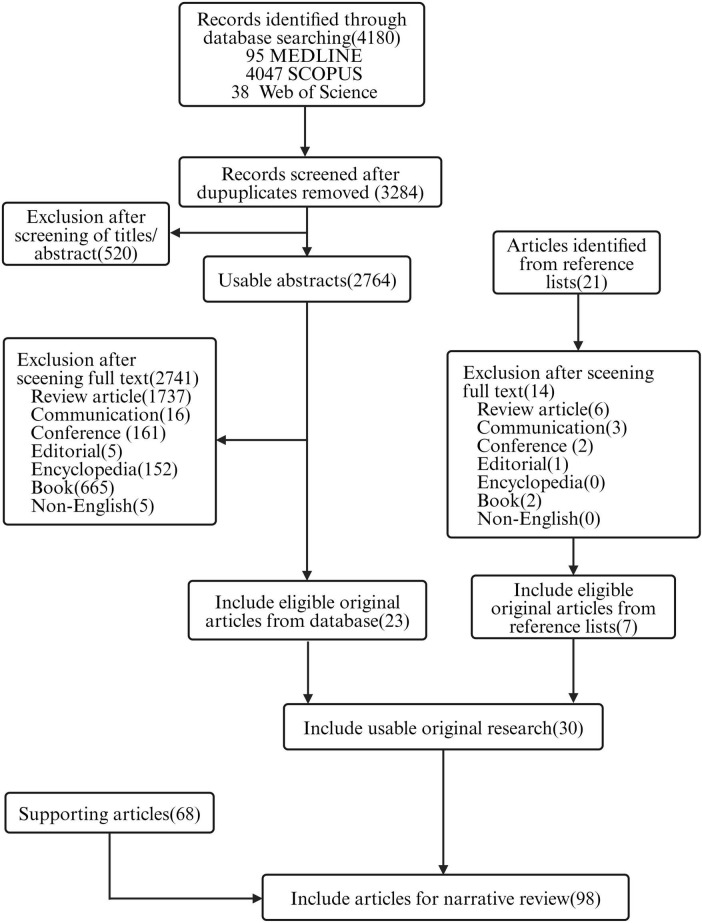
Flowchart of the review (created by BioRender.com, 2024).

**FIGURE 3:**
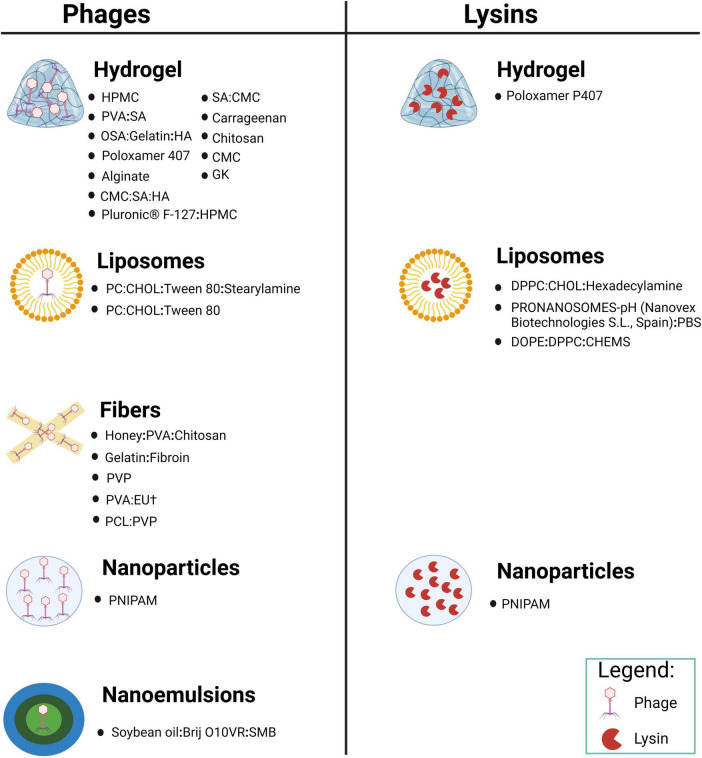
Novel delivery systems encapsulating phage and its encoded lysin in various materials prepared for the treatment of WIs (created by BioRender.com, 2024).

We conducted a relative comprehensive literature search of PubMed, Scopus and Web of Science, retrieving in total 4,180 articles, distributed as follows: 95 from PubMed, 4,047 from Scopus and 38 from Web of Science. Our methodologically rigorous and comprehensive screening process, as shown in Figure 2, provides the reader with a clear overview of the methodology and results. Through meticulously removing duplicates, filtering irrelevant article types and performing in-depth relevance screening of titles and abstracts, we obtained 30 research articles that were closely related to our review’s scope, as shown in the tables below (Kumari et al., 2010, 2011; Esteban et al., 2014; Hathaway et al., 2015; Chadha et al., 2017; Hathaway et al., 2017; Sarhan and Azzazy, 2017; Chhibber et al., 2018; Bai et al., 2019; Kaur et al., 2019; Portilla et al., 2020; Zhang et al., 2020; Beschastnov et al., 2021; Sarhan et al., 2021; Shen et al., 2021; Yan et al., 2021; Ilomuanya et al., 2022; Li et al., 2022; Morais et al., 2022; Mabrouk et al., 2022; Dehari et al., 2023; Kielholz et al., 2023; Mukhopadhyay et al., 2023; Shafigh Kheljan et al., 2023; Ilomuanya et al., 2024; Khazani Asforooshani et al., 2024; Narayanan et al., 2024; Shiue et al., 2024; Suchithra et al., 2024; Vacek et al., 2024). According to the currently available literature, novel encapsulated phage delivery systems for the treatment of WIs have been studied much more than lysins (Figure 3). To provide a broader perspective and enhance the organization of the review, we included 68 additional articles in our text.

## 4 Hydrogel

Hydrogels are the most commonly used carriers for delivery of biomolecules in tissue engineering. Hydrogels are highly hygroscopic and capable of retaining large amounts of water, making them suitable for phage delivery. They help protect the skin from excessive fluid loss while absorbing wound excretions (Merabishvili et al., 2017). Hydrogel formulations not only improve the hydration balance at the wound site, but also stabilize the phage by allowing hydrogen bond formation between water and phage proteins (Jones and Vaughan, 2005).

Recently, many animal studies have shown that hydrogels for delivering phages can be used for WIs with MDR bacteria (Table 2). The study implemented by Kumari et al. strongly suggested that hydrogels containing phage Kpn5 have a therapeutic role in the treatment of murine burn WIs (Kumari et al., 2011). Using a mouse model, a phage-loaded polyvinyl alcohol (PVA)-sodium alginate (SA) hybrid dressing was shown to display some characteristics of bacterial pathogen killing and promoting burn wound healing (Kaur et al., 2019). In a recent study of *P. aeruginosa*-infected rats with thermal skin injury, phage vB_Pae_SMP1 or vB_Pae_SMP5 or a biphage cocktail mixed with 5% w/v carboxymethylcellulose (CMC) hydrogel could control *P*. *aeruginosa* infections in burn wounds, promote dermal collagen fiber formation and accelerate healing at the site of injury, as evidenced by histopathological examination (Mabrouk et al., 2022).

**TABLE 2:** Summary study of hydrogel for phages and lysins in the topical management of WIs.

References	Application	Material(s)	Phage	Dosage concentration	Target bacteria	Study design	Major findings
**Phage-delivering hydrogel**							
Kumari et al. (2010)	Burn WIs	HPMC	Kpn5	10^8^ PFU/mL	*K. pneumonia*	*In vivo* (mice)	A single topical application of the phage rescued mice from *S. pneumoniae* B5055 infection compared to multiple applications of honey and aloe vera gel.
Kumari et al. (2011)	Burn WIs	HPMC	Kpn5	10^8^PFU/mL	*S. pneumoniae*	*In vivo* (mice)	A single topical application of Kpn5 phage rescued mice from *S. pneumoniae* B5055 infection and significantly reduced animal mortality.
Kaur et al. (2019)	Burn wound	PVA; SA	MR10	MOI 10	*MRSA*	*In vitro;* *in vivo* (mice)	The dual coated hydrogel membrane delivering both MR10 phage and minocycline proved to be better treatment strategy to treat the resistant burn wound infection rather than phage and antibiotic alone.
Zhang et al. (2020)	Wound damage	OSA; gelatin; HA	T7	10^7^PFU/mL	*E. coli*	*In vitro;* *in vivo* (mice)	An injectable hydrogel encapsulating aFGF and T7 can promote skin regeneration and prevent bacterial infections in mice, thereby promoting wound healing process.
Yan et al. (2021)	WIs	Poloxamer 407	IME-AB2	10^9^PFU/mL	*A. baumannii*	*In vitro*	The hydrogel formulation significantly inhibited microbial survival in an *in vitro* wound infection model using porcine skin (90% reduction in bacterial count after 4 h of treatment).
Shen et al. (2021)	Wound	Alginate	Phage HZJ	3 × 10^5^PFU/mL; 3 × 10^6^PFU/mL	*E.* .*coli*	*In vitro*	After phages were embedded in hydrogel, HZJ lysed 57–67% of bacteria within 2 h and the antibacterial effects lasted at least 24 h.
Beschastnov et al. (2021)	Wound	PVA; PBS; Lidocaine; Succinic acid	“Complex Pyobacterio phage” (Microgen, Russia)	NM	*Aureus*	*In vitro*	On days 1–3, no secondary growth was recorded in the phage-containing hydrogel.
Ilomuanya et al. (2022)	Chronic WIs	Chitosan	ΦAB140; ΦAB150	2.5 × 10^8^PFU/mL	*A. baumannii*	*In vitro;* *in vivo* (rat)	The application of particulate carrier technology provides lysogenic activity against *A. baumannii* while promoting wound healing.
Mabrouk et al. (2022)	Infected thermal wounds	CMC	vB_Pae_SMP1; vB_Pae_SMP5	10^8^PFU/mL	*P. aeruginosa*	*In vitro;* *in vivo* (rat)	Phage vB_Pae_SMP1 or vB_Pae_SMP5 or a biphage cocktail formulated as a hydrogel was able to control *P. aeruginosa* infections in burn wounds and promote wound healing while reducing animal mortality.
Shafigh Kheljan et al. (2023)	WIs	SA; CMC	PB10;PA19	3 × 10^7^(PB10); 4 × 10^7^PFU/mL(PA19)	*P.* .*aeruginosa*	*In vitro;* *in vivo* (mice)	Phage-containing hydrogels and antibiotic-containing hydrogels have almost the same antibacterial effect. There is a synergistic effect between cocktail phages and antibiotics.
Mukhopadhyay et al. (2023)	WIs	Pluronic^®^ F-127; HPMC	vB_AbaM-IME-AB2	10^9^PFU/mL	*A. baumannii*	*In vitro;* *in vivo* (porcine)	Phage-colistin-containing hydrogels effectively killed bacteria in both planktonic and biofilm states, and significantly reduced the bacterial burden in a pork skin wound infection model.
Dehari et al. (2023)	Burn wounds	Chitosan	BPSAΦ1; BPPAΦ1	4 × 10^9^ (BPSAΦ1); 1.3 × 10^4^ PFU/mL(BPPAΦ1)	*P. aeruginosa;* *S. aureus*	*In vitro;* *in vivo* (rat)	The incorporation of the phages in the mixed bacteriophage-gel protected the phages, sustained the phages release, improved the antibacterial activity, promoted the wound healing.
Narayanan et al. (2024)	Wound	Marine polysaccharide carrageenan	vB_Eco2571-YU1	10^10^PFU/mL	*E. coli*	*In vitro*	The long-term storage of hydrogels at 4°C and found that the titer value decreased during a time-dependent period of 28 days.
Vacek et al. (2024)	WIs	GK	phage 812K1/420	10^11^ PFU/mL	*MRSA*	*In vitro;* *in vivo* (porcine)	Phage-associated hydrogel has good biological properties, significant antimicrobial properties and promotes wound healing and re-epithelialization of infected wounds in pocrinne model.
Khazani Asforooshani et al. (2024)	WIs	CMC; SA; HA	phage EF-M80	10^7^ PFU/ml	*E. faecium*	*In vitro;* *in vivo* (mice)	The phage-loaded hydrogel has obvious antibacterial properties and can significantly promote the healing of infected wounds.
Shiue et al. (2024)	WIs	Alginate	Ph(S);Ph(E); Ph(P)	8.06 × 10^7^ [Ph(S)]; 7.24 × 10^7^[Ph(E)]; 2.94 × 10^7^ PFU/mL[Ph(P)]	*Salmonella enterica;* *E. coli;* *P. aeruginosa*	*In vitro;* *in vivo* (mice)	The repaired tissue in the phage-cocktail dressing group had new capillary vessels and no sign of inflammation in its dermis, and its epidermis had a higher degree of re-epithelialization.
**References**	**Application**	**Material(s)**	**Lsyin**	**Dosage concentration**	**Target bacteria**	**Study design**	**Major findings**
**Lysin-delivering hydrogel**							
Li et al. (2022)	WIs	Poloxamer P407	LysP53	500 μg/mL	*A. baumannii*	*In vitro*	LysP53 hydrogel could inhibit bacterial growth on a pig skin decolonization model.

NM, not mentioned.

The addition of other substances to hydrogels has similarly been demonstrated to have positive effects on wound healing. An injectable hydrogel encapsulating acidic fibroblast growth factor (aFGF) and phage had significant bacteriostatic activity against MDR *Escherichia coli* (*E. coli*), and significantly increased the VEGF, α-SMA and collagen I expression levels in regenerated tissues at day 7, indicating that hydrogels containing aFGF can effectively improve tissue regeneration, enhance angiogenic activity, promote skin regeneration and prevent bacterial infections (Zhang et al., 2020). Moreover, the use of phage preparations in combination with anaesthetics may provide a painless solution for wound treatment. Beschastnov et al. (2021) produced hydrogel wound dressings with the optional addition of lidocaine. The results of *in vitro* studies showed that phages immobilized in hydrogels retained their viability and solubilizing activity, and phage activity was maintained when combined with lidocaine.

Notably, phages were well tolerated and remained stable in hydrogels formulated with neutral polymers (Chang et al., 2020; Kim et al., 2021). At physiological pH, the caps of phages exhibit a net negative charge, whereas the tails exhibit a net positive charge (Anany et al., 2011). Anionic polymers will interact with the positively charged phage tails by electrostatic attraction, blocking the binding of the tail fibers to the host bacterial receptor, resulting in the phage no longer being infectious to the host bacteria. Osborne et al. (2018) prepared phage-containing hydrogels using nonionic and anionic polymers. Phages formulated in carbomer (anionic polymer) hydrogels displayed a 99.95% decrease in titre after 4 weeks of storage at 4°C, whereas phages formulated in hydroxyethylcellulose gel (nonionic) hydrogels remained biostable (Osborne et al., 2018). The limited number of studies mentioned above highlights the importance of selecting neutral polymers that minimize charge-induced phage inactivation and thus maintain phage stability. Phages PEV1 and PEV31 remained stable in aqueous gels formulated with poly(ethylene oxide) (PEO), PVA and hydroxypropyl methylcellulose (HPMC) (Chang et al., 2021). Due to the limited number of hydrogels tested for phage stability, neutral polymer hydrogels such as guar gum, agarose, polyvinylpyrrolidone or polyacrylamide can be investigated in the future.

Currently, there is a lack of research in the field on how to maintain the stability of phage hydrogels for long-term storage. The stability of phages depends on many of the following factors: pH, temperature, formulation composition, and light (Jonczyk-Matysiak et al., 2019). There are only a few studies on the short-term storage of hydrogel to maintain the stability of phages. The stability of *Klebsiella* phage Kpn5 in a 3% HPMC hydrogel at 37°C was only seven days (Kumari et al., 2010, 2011). *Klebsiella* phage Kpn5, *Pseudomonas* phage PA5 and *Staphylococcus* phage MR10 remained active after 28 days of storage at room temperature in PVA-SA hydrogels (Kaur et al., 2019). It is clear that maintaining long-term phage stability in hydrogels deserves more work to improve.

Hydrogels are also an effective delivery system for sustained lysin release to the site of infection, as illustrated in Table 2. A thermosensitive hydrogel containing the G^–^ lysin LysP53 was safe, non-cytotoxic and hydrolysed the resting *Acinetobacter baumannii (A. baumannii)* peptidoglycan, resulting in bactericidal activity (Li et al., 2022). LysP53-loaded hydrogels may provide a novel solution for the treatment of *A. baumannii*-induced WIs (Li et al., 2022). Thermostable polymers should be considered for investigation. Following infection, the skin temperature increases, which has been observed in chronic leg ulcers. A cocktail consisting of cysteine, the CHAP structural domain of phage K lysin and lysostaphin was added to a poly (N-isopropylacrylamide) (PNIPAM) gel (Hathaway et al., 2017). An increase in temperature successfully triggered their release. In addition, the lysin-loaded hydrogel had the properties of killing pathogenic bacteria in fracture-associated infections and promoting fracture healing. Yao et al. (2021) designed a chimeric ClyC-loaded alginate hydrogel (ClyC-AH), which was non-cytotoxic and retained the stability and activity of ClyC, while providing sustained ClyC release and antimicrobial efficacy against *S. aureus*. In a mouse model of osteomyelitis, ClyC-AH reduced the number of *S*. *aureus* in the femur and surrounding tissues. However, none of these studies had yet assessed the long-term storage stability of lysin in hydrogels, which is essential for the development of commercially viable lysin products. This shows that it is a major challenge to maintain the long-term stability of lysin in hydrogels.

## 5 Liposomes

Liposomes are spherical particles consisting of natural lipid bilayers that can contain drugs; hydrophobic or amphiphilic molecular drugs are present within the membrane or at the interface, respectively (Sercombe et al., 2015; Kuhn et al., 2017). Liposomes can be prepared as multi- or unilamellar vesicles of various sizes. The non-immunogenicity, biodegradability and biocompatibility of liposomes make them a suitable phage delivery system (Colom et al., 2015).

A summary of studies on the application of liposome delivery systems encapsulating phage for WIs is presented in Table 3. Liposomes help to improve phage survival, stability and retention time at the site of WI. Chadha et al. (2017) evaluated the potential of liposomes as phage delivery vehicles for the treatment of burn WIs. The results showed that liposome delivery of phages increased phage retention time in the body and enhanced efficacy compared to phage use alone, which in turn resulted in greater bacterial volume reductions in the bloodstream and major organs of mice treated with the contained cocktail of phage liposomes, and consequently a more rapid rate of infection regression. Diminished phage immunogenicity reduced cytokine levels (interleukin-1β and tumor necrosis factor-α) compared to baseline (Chadha et al., 2017). In addition, liposomes protect phages from pH-induced inactivation, which is particularly important in acidic environments such as wound infection sites. Chhibber et al. (2018) evaluated the ability of a liposome-encapsulated cocktail of phages to resolve diabetic excisional WIs induced by *S. aureus*. The results of *in vitro* stability studies and *in vivo* phage titre assays indicated that WI treatment with phage-loaded liposomes improved phage persistence *in vitro* and *in vivo*, suppressed bacterial counts and inflammation levels in wound tissue and promoted wound tissue collagen fiber maturation, improving wound healing.

**TABLE 3:** Summary study of liposomes for phages and lysins in the topical management of WIs.

References	Application	Material(s)	Phage	Dosage concentration	Target bacteria	Study design	Major findings
**Phage-delivering liposomes**							
Chadha et al. (2017)	Burn WIs	PC;CHOL; Tween 80; Stearylamine	KØ1; KØ2; KØ3;KØ4; KØ5	10^8^PFU/ml	*Pneumoniae*	*In vivo* (mice)	The use of phage cocktail-loaded liposomes reduced the amount of bacteria in the blood and major organs of mice and prevented the death of test animals.
Chhibber et al. (2018)	Diabetic wound infection	PC;CHOL; Tween-80	MR-5; MR-10	10^9^PFU/ml	*S. aureus*	*In vitro; in vivo* (mice)	Phage-loaded liposomes increase the duration of phage action at the wound site, effectively inhibit the growth of wound bacteria and promote the healing of infected wounds.
**References**	**Application**	**Material(s)**	**Lysin**	**Dosage concentration**	**Target bacteria**	**Study design**	**Major findings**
**Lysin-delivering liposomes**							
Bai et al. (2019)	Bacteria infection	DPPC; CHOL; hexadecylamine	BSP16Lys	NM	*Salmonella Typhimurium; E. coli*	*In vitro*	BSP16Lys-loaded liposome treatment reduced cell counts of *Salmonella typhimurium* and *E. coli* cells by 2.2 log CFU/mL and 1.6 log CFU/mL, respectively.
Portilla et al. (2020)	Bacteria infection	PRONANOSOMES-pH (Nanovex Biotechnologies S.L., Spain); PBS	LysRODI	40μg/mL	*S. aureus*	*In vitro*	Encapsulated endolysin was effective in reducing the number of *S. aureus* cells in floating cultures and biofilms by greater than 2 log units.
Morais et al. (2022)	MDR bacteria infection	DOPE; DPPC; CHEMS	Pa7;Pa119	6.25μg/mL	*P*. aeruginosa	*In vitro*	Application of encapsulated lysin to a model of *P. aeruginosa* revealed a decrease in bacterial cell viability and cell lysis

NM, not mentioned.

Not to be overlooked is the fact that cationic liposome are well suited for delivery of phages. Cationic liposomes can also be used to enhance interactions with negatively charged bacteria, because there is a higher affinity between positively charged liposomes and bacteria. In addition, cationic liposomes have mucoadhesive properties that prolong the drug retention time at the administration site, resulting in controlled release and improved therapeutic efficacy (Shaikh et al., 2011). A myxoma virus and two Podoviridae viruses did not degrade when delivered in cationic liposome particles, thus prolonging their residence time in animals (Colom et al., 2015). The obstacles observed so far, such as low encapsulation efficiency, difficulty in controlling liposome size, and loss of active phage during preparation, suggest that liposomes are not perfect delivery vehicles. Therefore, rigorous testing is needed to determine the general suitability of delivery vectors, i.e., whether liposomes can be used and which types of lipids may be suitable.

At the same time, alternatives to liposomes, such as so-called transferosomes, i.e., liposomes containing detergents, must be explored. Transferosomes are cationic liposomes consisting of phosphatidylcholine, tween-80 and stearyl amine, which are used to improve permeability (Chhibber et al., 2017). Chhibber et al. (2017) evaluated the use of transferosomes as a transdermal delivery system for encapsulating *MRSA* phage cocktails. The results of *in vitro* stability and *in vivo* phage titre experiments showed that the transporter-delivered cocktail phage had better persistence and stability compared to free phage. Thigh infections in rats treated with transposome-delivered cocktail phage resolved within 7 days, compared to 20 days in untreated animals. In addition, this study found that transposome-delivered phage had better therapeutic advantages over free phage in the treatment of skin and soft tissue infections. Unlike free phage, the transferosome-delivered phage cocktail protected all test animals (no deaths) even when administered with a 12-hour delay after infection. However, Niosomes composed of nonionic surfactants and other amphiphilic molecules as well as cholesterol face similar challenges as all amphiphilic vesicle particles (Marianecci et al., 2014).

Lysin-loaded liposomes are also a very attractive option. To date, there are three examples of liposome-delivered phage lysins, as summarized in Table 3. For example, Hajiahmadi et al. (2019) prepared a number of vancomycin-loaded nanoliposomes conjugated with anti-*staphylococcal* proteins (lysozyme) and evaluated their *in vitro* and *in vivo* efficacy as topical therapy for *MRSA*. Targeted liposomes inhibited bacterial infections *in vitro* and *in vivo* more effectively than non-targeted vancomycin liposomes. The results suggest that a novel nanocarrier (lysostaphin-conjugated coupled liposomal vancomycin) can be used as an optimal topical antibacterial construct for the treatment of *MRSA* skin infections (Hajiahmadi et al., 2019). Here, Portilla et al. (2020) also found that pH-sensitive liposomes can be used to deliver the endolysin LysRODI, which eliminates *S. aureus* planktonic and biofilm populations in weakly acidic environments. This included different human bodily regions, such as the skin or vagina, as well as certain food or surgical sites where staphylococcal contamination may be a problem. Notably, *S. aureus* biofilm formation is enhanced at ambient pH values between 4.75 and 5.5, which is the range of content release of the liposomes used in the study performed by Foulston et al. (2014). Nevertheless, although liposome-encapsulated LysRODI was effective against biofilm cells, its activity was significantly hampered by the biofilm matrix compared to the free lysin. More research in the future should overcome it by altering the composition of lipid vesicles or trying combinatorial strategies with matrix-degrading enzymes. Another interesting strategy to explore wounding is to dope the surface of liposomes with antibodies or other proteins that can interact with biofilm molecules and thus facilitate the penetration of lipid vesicles into these robust structures (Portilla et al., 2020).

## 6 Electrospun fibers

In addition to the use of liposomes, phages can be delivered in electrospun fibers. Phages can withstand an electric field of up to 40 kV/cm for 5 min and can be embedded in fibers during the electrospinning process, and a variety of materials (cellulose diacetate, PEO, PVP, polyester urea) have been demonstrated to be suitable for this method (Korehei and Kadla, 2013; Dai et al., 2014; Korehei and Kadla, 2014; D rehei and Kadla). The key point is that a large number of synthetic agents (anticancer, anti-inflammatory and antimicrobial drugs), bioactive agents (DNA, proteins, enzymes) and living cells (viruses, algae) can be doped during the electrostatic spinning process to develop electrospun fibers that can be further used as suitable carrier systems (Kaur et al., 2021).

Fiber production may expose the phage particles to damage. Loss of phage viability during electrostatic spinning due to rapid solvent evaporation and drastic changes in osmotic pressure is found. While fibers can still be produced using pure distilled water, such compositions are not ideal for electrospinning, phage delivery or long-term storage. By contrast, the morphology produced by fibers composed of buffer solutions has been shown to provide a thermodynamically favorable microenvironment in which phages will be encapsulated, thus maintaining their infectivity for up to 8 weeks (Dai et al., 2014). Furthermore, the addition of sugars (e.g., tohalose) or solvent components can stabilize phages and reduce the formation of salt crystals, thus preventing complete phage inactivation (Dai et al., 2014). Despite the protective effect of the addition of alginate, buffer on the high pressure and spinning process, partial loss of phage activity was observed (Zussman, 2011; Dai et al., 2014).

Studies of phage-loaded electrospun fibers applied to WIs are summarized in Table 4. There are two general types of electrostatic spinning: emulsion and coaxial electrostatic spinning. These two types of electrostatic spinning can solve the disadvantages of electrostatic spinning mentioned above. Emulsion electrospinning involves pre-encapsulating phage in alginate nanoparticles, freeze-drying them, suspending them in a polymer-containing solvent and electrostatically spinning them. Doping of emulsion electrospun fibers containing T4 phage into alginate capsules provided a shielding barrier against rapid dehydration stress, and only a slight decrease in phage activity was observed (Korehei and Kadla, 2013). Phage-loaded gelatine/silk fibroin fibers prepared by Sarhan et al. had an amorphous structure, good thermal stability at temperatures up to 300°C, good biocompatibility, promoted fibroblast proliferation and displayed significant inhibition of MDR *P*. *aeruginosa* (Sarhan et al., 2021). This suggested that the electrospun fibers are capable of maintaining phage stability, and are a promising dressing for MDR *P. aeruginosa* infected wounds (Sarhan et al., 2021). In contrast, coaxial electrospinning involves incorporating phage in the core of a core-shell fiber structure, thereby encapsulating the phage in the core-shell fiber. Coaxial spinning produced a continuous core/shell morphology with a more homogeneous distribution of phage in the fiber core and no major changes in the permeation environment, thus maintaining full solubilization activity (Korehei and Kadla, 2013). Kielholz et al. (2023) employed a coaxial electrostatic spinning technology approach to fabricate functional fibers with phage core/PVP shells with mechanical properties that are well suited for *P*. *aeruginosa* and *S*. *aureus*-infected wound applications. This core-shell fiber has good rapid-release kinetics, biocompatibility and antimicrobial activity. On the basis of the above features, this fiber prepared by coaxial electrostatic spinning is suitable for WIs (Kielholz et al., 2023). Unfortunately, the storage time of these phage-loaded electrospun fibers was short, with the antimicrobial properties remaining unchanged for 90 days at 4°C and 60 days at 25°C. These factors limit their wide clinical application (Kielholz et al., 2023; Ilomuanya et al., 2024).

**TABLE 4:** Summary study of other novel delivery systems for phages and lysins in the topical management of WIs.

References	Application	Material(s)	Phage	Dosage concentration	Target Bacteria	Study Design	Major findings
**Electrospun fibers**							
Sarhan and Azzazy (2017)	Wound	Honey; PVA; Chitosan	PS1	10^8^–10^9^ PFU/mL	*P. aeruginosa*	*In vitro*	Phage-loaded nanofibers almost completely killed MDR *P. aeruginosa*.
Sarhan et al. (2021)	Wound	Gelatin; fibroin	Phg	10^8^–10^9^ PFU/mL	*P. aeruginosa*	*In vitro*	The number of *P. aeruginosa* was reduced by 2 logarithmic values in the Phage-loaded nanofiber group and by 4 logarithmic values in the bacterial counts after 16 h as compared to the nanofiber alone and the negative control group.
Kielholz et al. (2023)	WIs	PVP	JG004;EBHT	NM	*S.* .*aureus; P. aeruginosa*	*In vitro*	Phage-loaded core/shell nanofibers exhibit bacteriostatic activity against *S. aureus and P. aeruginosa.*
Suchithra et al. (2024)	WIs	PVA; EU†	RuSa1; PseuPha1	10^12^PFU/mL (RuSa1; seuPha1)	*P.* .*aeruginosa; S. aureus*	*In vitro; in vivo* (mice)	The sustained release, quick wound closure, declined abundance of pathogenic bacteria in wound site and histopathological signs of recovery corroborated the therapeutic efficacy of the phage-loaded fibers.
Ilomuanya et al. (2024)	WIs	PVP; PCL	PΦ-Mi-Pa 51; PΦ-Mi-Pa 120; PΦ-Mi-Pa 133	10^8^(PΦ-Mi-Pa120; PΦ-Mi-Pa133);10^7^PFU/mL (PΦ-Mi-Pa051)	*P. aeruginosa*	*In vitro; in vivo* (rats)	Phage-loaded fibers displays antibacterial activity and promotes wound healing.
**References**	**Application**	**Material(s)**	**Phage/lysin**	**Dosage concentration**	**Target bacteria**	**Study design**	**Major findings**
**Nanoparticles**							
Hathaway et al. (2015)	WIs	PNIPAM	Phage K	10^9^ PFU/ml	*S. aureus*	*In vitro*	Phage-containing PNIPAM-co-ALA nanospheres successfully lysed *S. aureus* ST228 at 37°C, whereas bacterial growth was unaffected at 25°C.
Hathaway et al. (2017)	Skin and soft tissue infections	PNIPAM	Endolysin CHAP(K)	1 to 64 μg/ml	*S. aureus*	*In vitro*	Bacteria were lysed by the sustained release of endolysin CHAP(K) and lysostaphin from PNIPAM nanoparticles at a skin temperature of 37°C, whereas bacteria maintained growth at a skin temperature of 32°C in uninfected skin.
**References**	**Application**	**Material(s)**	**Phage**	**Phage dosage**	**Target bacteria**	**Study design**	**Major findings**
**Nanoemulsions**							
Esteban et al. (2014)	Wound	Soybean oil; Brij O10VR; SMB	Phage K	10^6^-10^8^ PFU/ml	*S. aureus*	*In vitro*	Compared to phage liquid alone, phage K-loaded nanoemulsions have stronger antimicrobial activity and can rapidly kill three different strains of *S*. *aureus.*

NM, not mentioned.

By contrast, little research has been published on the application of fiber delivery systems encapsulating phage-derived lytic lysins for infected wounds. Taking inspiration from nature, Miao et al. (2011) used lysostaphin (Lst), a cytolytic enzyme with specific bactericidal activity against *S. aureus*, to create an anti-infective bandage. Lst was immobilized in a cellulose binding module consisting of the room temperature ionic liquid 1-ethyl-3-methylimidazolium acetate electrospun cellulose, cellulose-chitosan and cellulose-poly(methyl methacrylate) in homogeneous solution to generate biocompatible fibers. The fibers were chemically treated to generate aldehyde groups for covalent immobilization of Lst. The Lst-functionalised cellulose fibers obtained from the processing could be made into bandage formulations, which displayed antistaphylococcal activity against *S. aureus* in an *in vitro* skin model and low toxicity for keratinocytes, suggesting that these materials are biocompatible and have a good potential to serve as an antimicrobial matrix in wound healing applications. This approach provides a new idea for the development of a fiber delivery system encapsulating phage-encoded lysins.

## 7 Nanoparticles

Recent advances in nanotechnology have opened new frontiers for the application of small molecules, proteins, viruses and other pharmaceutically active substances for chronic wound healing (Peng et al., 2022; Liu et al., 2023; Wang et al., 2023). The size and physicochemical properties of nanostructures allow intracellular delivery of actives, their protection from degradation and their enhanced penetration into wounds. In addition, delivery of active substances in nanocarriers enables different drug release profiles, thus meeting the requirements of wound healing (Liu et al., 2023).

Nanoparticles are normally small-sized nanospheres constructed at the molecular level. These spheres can be used to deliver actives drug that degrade in the environment. Phage delivery processes need to be carefully designed to prevent damage to the viral capsid and DNA/RNA components, and to prevent a decrease in phage activity during production. Hathaway et al. (2015) developed nanospheres consisting of PNIPAM, to which *S*. *aureus* phage K was incorporated, as shown in Table 4. When the temperature rises (usually at the site of bacterial skin infection), the nanospheres dissolve, releasing active phage cargo, which inhibits bacterial growth (Hathaway et al., 2015). Hyaluronic acid (HA) methacrylate is coated on the agarose gel containing *S. aureus* phage K, essentially creating a two-layer hydrogel. During infection, the outer layer of HA is solubilized by enzymes produced by the pathogen, and phages are released in the vicinity of the infection (Bean et al., 2014).

Nanoparticle delivery of lysins protects the proteins from degradation, provides sustained release and aids in achieving better bacteriolysis. Kaur et al. (2020) evaluated the antibacterial characteristics of alginate-chitosan nanoparticles (Alg-Chi NPs) loaded with LysMR-5 against *S. aureus*. Alg-Chi Nps loaded with LysMR-5 displayed enhanced bactericidal activity against *S. aureus* relative to single Alg-Chi Nps. The nanoparticle diameters of the LysMR-5-loaded formulations remain essentially unchanged for about 9 weeks at 4°C, but will change significantly at 25°C (Kaur et al., 2020). This shows that the long-term stability of nanoparticle formulations still requires us to be worthy of vigilance. Hathaway et al. (2017) delivered the truncated endolysin CHAP(K) and bacteriocin lysostaphin within PNIPAM nanoparticles, which at the specific temperature of 37°C release and treat skin infections caused by *S. aureus*, as shown in Table 4. In addition, the combination of CHAP(K) and bacteriocin lysostaphin acted synergistically to accelerate control and the response time to MRSA treatment (Hathaway et al., 2017). The use of nanoparticles as delivery vehicles for lysins is a novel approach in making lysins a viable treatment for infections by utilizing and developing the physical and chemical properties of new and existing nanoparticles.

## 8 Nanoemulsions

Nanoemulsions are colloidal spherical systems with amorphous and lipophilic (negatively charged) surfaces of average particle sizes between 10 and 1,000 nm (Jaiswal et al., 2015). Nanoemulsification technology allows for the formation of homogeneous nanoemulsions by adding a mixture of microorganisms, cells, enzymes and drugs, as well as compatible polymers to the oil, and then further dispersing the mixture into another phase of vegetable oil (McClements and Rao, 2011). The nanoemulsion may be water-in-oil (W/O), but may also be oil-in-water (O/W), and in some cases a third phase may be present (Puapermpoonsiri et al., 2009; Esteban et al., 2014). Nanoemulsions can be further stabilized by the addition of emulsifiers and stabilizers. Therefore, nanoemulsions provide a new way to encapsulate sensitive molecules such as proteins, enzymes and phages, among others, in a nanoporous matrix. Nanoemulsion formulations can facilitate transdermal absorption by varying droplet size, altering emollients and/or emulsifiers and adding particulate components to the mixture (Otto et al., 2009). This makes them particularly effective in treating deep skin infections.

Multiple emulsions prepared by microfluidic methods, such as W1/O/W2 emulsions (where the intermediate phase is immiscible with the inner and outer phases), have a high degree of precision and controllable droplet size and shape (Dickinson, 2011). Depending on the number of immiscible phases, different amounts of multiple active ingredients can be prepared (e.g., phage co-encapsulated with antibiotics or phage co-encapsulated with antimicrobial peptides or lysin), with the release sequence selected as desired. Such multishell droplets can maximize the protection of highly sensitive phages or phage enzymes from external stressful environments. For example, the outer shell protects the phage from the pH of gastric acid because it is acid-resistant. The next layer of the inner shell may subsequently be used for burst release to act immediately in releasing a large number of phages for immediate bacterial population control. The final layer or inner shell can be used for sustained release of phages or antibacterial substances to maintain a slow but sustained release pattern, which is particularly important for treating chronic infections, and also reduces or avoids the need to reuse phages or antibacterial substances.

Nanoemulsions can be considered to be a new reliable option for phage delivery. To provide maximum protection to highly sensitive phages from external stressful environments, nanoemulsification technology can deliver single or multiple active ingredients with sequential release, depending on the desired regimen (Kaur et al., 2021). Esteban et al. (Esteban et al., 2014) used nanoemulsion encapsulation to stabilize phage K lysates, which were subsequently tested *in vitro* against three species of *S. aureus*, as shown in Table 4. Here, the phage surrounded the nanodroplets instead of being encapsulated in them. Phages in the emulsion had higher activity than unemulsified phages over a period of 10 days at 4°C and room temperature. Phage emulsion formulations were also effective in killing three *S. aureus* strains within 2–5 h relative to liquid phage controls. Because both phages and bacteria have a very negative “charge,” the presence of nanoemulsions reduces the electrostatic repulsion between them and facilitates their interaction, thus enhancing the anti-microbial or killing effect (Esteban et al., 2016). In addition, due to less surfactants required in the preparation process, such O/W nanoemulsions are more permeable transdermally and more biocompatible with skin tissues (Azeem et al., 2009). Nanoemulsified phage formulations are used for superficial and deep skin infections as well as wound application/dressings, resulting in sustained active phage release. However, the activity of the phage emulsion did not change significantly over a 10-day period, whereas the lysogenic activity of the phage appeared to be erratic, with different results for each day of treatment and each storage temperature (Esteban et al., 2014). To further deepen the research on nanoemulsion formulations for delivery of phage, the above issues need to be thoroughly addressed.

Strategies to enhance antimicrobial efficacy through nanoemulsions also include the incorporation of lysin into antimicrobial matrices and systems that extend the duration of lysin administration. One approach to prolong lysin administration at the site of infection is nanoemulsion delivery of lysin. These thermodynamically stable particles adhere well to the skin, which improves bioavailability (Tayeb and Sainsbury, 2018). Recombinant lysostaphin has been formulated in a nanogel to extend the release time up to 8 h. A treatment regimen of three topical applications per day was sufficient to cure *MRSA*-infected abscesses in mouse skin (Nour El-Din et al., 2020). Although lysozyme is a bacteriocin, similar applications could be envisaged using phage-derived or engineered lysin.

## 9 Discussion

The treatment of MDR bacterial WIs raises many challenges and new insights are urgently needed in this field (Raducu et al., 2024). Phage and lysin formulations may be a promising strategy to treat MDR bacterial WIs (Moghadam et al., 2024; Wang et al., 2024). Novel delivery systems (e.g., hydrogel, liposomes, electrospun fibers, nanoparticles, nanoemulsions) in combination with phages or lysins provide a rich toolbox to treat MDR bacterial WIs. This article focuses on a review of some recent advances in novel delivery systems that deliver phages or lysins for topical application to WIs.

A good drug formulation must be able to encapsulate a specific drug and protect it from the surrounding environment, such as pH and temperature, among others, while maintaining the biological activity of the drug, particularly its antimicrobial properties (Rosner and Clark, 2021; Briot et al., 2022). In addition, the chosen drug delivery system must be biocompatible and capable of releasing the drug in an orderly and slow manner at the desired infection site (Rosner and Clark, 2021; Briot et al., 2022).

Existing literature on novel delivery systems encapsulating phages and lysins remains scarce, particularly on novel drug delivery systems encapsulating lysins. Phages are mostly administered as suspensions or, in the case of skin infections, associated with dressings (Pinto et al., 2020; Pinto et al., 2021). Most of the novel drug delivery systems regarding encapsulated phages were developed during just more than the last decade. It is of note that novel drug delivery systems for lysins started in 2017 (Pinto et al., 2021).

Recently, novel delivery systems encapsulating phages or lysins, such as hydrogel, liposomes, electrospun fibers, nanoparticles and nanoemulsions, have been reported, and these studies have confirmed that novel delivery systems encapsulating therapeutic substances are beneficial for healing infected wounds (Lin et al., 2024). However, each novel delivery system has its own limitations and challenges, such as the complexity of its manufacture, the potential loss of antimicrobial activity during processing, and the difficulty of stability of the formulation over long periods of storage. In addition, these studies rarely mention in detail the interactions between carriers and drugs and the level of comparison with other types of systems. Consequently, there are no clear conclusions as to which drug delivery system performs the best.

To date, various *in vitro* and *in vivo* studies have demonstrated the great therapeutic potential of novel delivery systems encapsulating phages or lysins for the treatment of WIs, and most of these studies have demonstrated their therapeutic benefit through animal models. However, few studies have considered the irregular shape and varying depths of clinical infected wounds; animal models of infected wounds are normally a simple and regular situation.

Despite the amount of research focusing on the therapeutic potential of novel drug delivery systems, there remains many practical challenges in treating patients with WIs. Because of the current gaps, greater attention needs to be paid to clinical specifics from different perspectives. First, infected wounds in patients are frequently associated with uncontrolled hyperglycaemia or vascular pathology (Liu et al., 2023). Ideally, novel delivery systems should encapsulate drugs that can lower blood glucose or ameliorate vasculopathy, in addition to phages or lysins. Second, the relationship between the action of the loaded drug and the pathological stage of the infected wound should also be elaborated. In addition, the strains in chronic infected wounds are diverse and change dynamically over time (Nahid et al., 2021). Because no single drug works optimally or is suitable for all types of infected wound conditions, future studies may also consider the development of novel drug delivery systems loaded with multiple active drugs to provide synergistic effects at different stages and for varying pathological tissue types as well as in multi-strain infections. Preparation techniques for novel drug delivery systems are also important, and these should be scalable, reproducible and cost-effective to allow their use for a wide range of clinical applications. Clinical validation of these drug delivery systems requires more extensive in *vivo* studies and clinical trials to confirm their safety, efficacy and performance in the real world.

## 10 Conclusion

Currently, WIs associated with MDR bacteria pose many difficulties. In the future, phage and lysin therapies will remain a popular research topic for the treatment of MDR-infected wounds. Phage and lysin formulations have evolved from traditional liquid formulations to novel delivery systems that have been demonstrated in different laboratory models. Limited experimental data suggest that novel delivery systems such as hydrogel, liposomes, electrospun fibers, nanoparticles and nanoemulsions encapsulating phages and lysins for the treatment of WIs have significant bacteriostatic properties and promote wound healing. Continuous improvement and innovation of novel drug delivery systems could bring closer the full realization of the therapeutic potential of phages and lysins, ultimately leading to improved wound care strategies and better patient outcomes for MDR bacterial WIs.
